# Shigellosis outbreak among persons experiencing homelessness—San Diego County, California, October–December 2021

**DOI:** 10.1017/S0950268823001681

**Published:** 2024-10-23

**Authors:** Elizabeth C. Ohlsen, Kristen Angel, Azarnoush Maroufi, Annie Kao, Maria J. Victorio, Lynnie S. Cua, Akiko Kimura, Kayla Vanden Esschert, Naeemah Logan, Temet M. McMichael, Mark E. Beatty, Seema Shah

**Affiliations:** 1 Epidemic Intelligence Service, San Diego, CA, USA; 2 Centers for Disease Control and Prevention, Atlanta, GA, USA; 3 County of San Diego Health and Human Services Agency, San Diego, CA, USA; 4 California Department of Public Health, Richmond, CA, USA; 5 University of California San Diego, San Diego, CA, USA

**Keywords:** community outbreaks, dysentery (bacillary)hand hygiene, hygiene-personal, Shigella

## Abstract

During October 2021, the County of San Diego Health and Human Services Agency identified five cases of shigellosis among persons experiencing homelessness (PEH). We conducted an outbreak investigation and developed interventions to respond to shigellosis outbreaks among PEH. Confirmed cases occurred among PEH with stool-cultured *Shigella sonnei*; probable cases were among PEH with *Shigella*-positive culture-independent diagnostic testing. Patients were interviewed to determine infectious sources and risk factors. Fifty-three patients were identified (47 confirmed, 6 probable); 34 (64%) were hospitalised. None died. No point source was identified. Patients reported inadequate access to clean water and sanitation facilities, including public restrooms closed because of the COVID-19 pandemic. After implementing interventions, including handwashing stations, more frequent public restroom cleaning, sanitation kit distribution, and isolation housing for ill persons, *S. sonnei* cases decreased to preoutbreak frequencies. Improving public sanitation access was associated with decreased cases and should be considered to prevent outbreaks among PEH.

## Introduction

Shigellosis is a gastrointestinal illness caused by *Shigella,* a genus of gram-negative, rod-shaped bacteria that are most frequently transmitted through a faecal–oral route. Shigellosis is the second leading cause of diarrhoeal mortality worldwide [1]. Approximately 80% of laboratory-confirmed cases of shigellosis reported in the United States are caused by *Shigella sonnei.* *Shigella* spp. can be transmitted through contaminated food, contaminated water, contaminated surfaces, or sexual activity [2]. In the United States, groups at the highest risk for shigellosis include children under the age of five years, persons experiencing homelessness (PEH), international travellers, and men who have sex with men (MSM) [3]. PEH often live in encampments, crowded shelters, or unsheltered conditions and have limited access to clean water and sanitation services, increasing the risk for *Shigella* transmission. *Shigella* species require an infectious dose of as low as 10 organisms to cause disease, contributing to ease of transmission, particularly in settings with limited access to clean running water [4].

Outbreaks of *S. sonnei* or *Shigella flexneri* among PEH have been reported since 2014 on the West Coast of North America, including outbreaks in Vancouver, British Columbia [5, 6]; Portland, Oregon [7]; Long Beach, California [8]; San Francisco, California [9]; and Seattle, Washington [10]. All were thought to be partially associated with limited access to clean water and sanitation. Findings from the 2015 Oregon outbreak demonstrated an increase in shigellosis infections among PEH, but not among housed persons, during periods of heavier precipitation. More rain might have exacerbated poor sanitary conditions, crowding in shelters and encampments, and waterborne transmission through contamination of untreated drinking water [7].

During October 2021, routine enteric disease surveillance and case investigation by the Epidemiology Unit of the Public Health Services department in the County of San Diego (SDC) Health and Human Services Agency identified five cases of shigellosis reported among PEH. All were *S. sonnei* with similar resistance patterns. An outbreak investigation was initiated because of the rapid increase of identified *S. sonnei* cases within a population at high risk for transmission and severe disease. The investigation objectives were to identify modifiable risk factors and possible sources of *S. sonnei* among PEH in SDC in order to reduce community transmission.

## Methods

### Initial investigation

Shigellosis is a reportable disease in California. The SDC Epidemiology Unit conducts laboratory-based surveillance of *Shigella* and other pathogens as directed by California State Public Health shigellosis guidance [11]. Monthly case counts are compared with average case counts from the preceding 30 years. During fall 2021, the number of *Shigella* isolates increased above the 30-year seasonal trend in SDC. Available demographic data suggested that PEH were disproportionately affected. The investigation team confirmed this by comparing monthly case counts during August–December 2021 in PEH and persons not experiencing homelessness (non-PEH) to 30-year monthly averages. An increase in cases among non-PEH was also noted. To improve case finding, a health alert was issued to registered clinicians through the California Health Alert Network in SDC to raise awareness and encourage testing for *Shigella* among PEH and others with diarrhoeal symptoms, and the public was encouraged through press releases to seek care for diarrhoeal symptoms.

### Outbreak case definition

We defined a confirmed case as occurring in a PEH residing in SDC with symptom onset on or after 16 August 2021 who had isolation of *S. sonnei* from a stool culture. Antimicrobial sensitivities and whole genome sequencing (WGS) of isolates were tracked but not included in the case definition. We defined cases as occurring in PEH if the patient self-identified during the case investigation interview or their medical records indicated they were unhoused or residing in a shelter for unhoused persons. Clinically compatible cases among PEH in SDC for which a culture-independent detection test (CIDT) was positive for *Shigella* species during the same period were considered probable cases. Non-PEH SDC residents with laboratory isolation of *S. sonnei* from a stool culture or who were CIDT positive for *Shigella* species and had symptom onset on or after 16 August 2021 were also tracked to identify possible spread outside of PEH groups but were not counted as outbreak cases.

### Interview process

Medical records from emergency department visits and hospitalisations associated with confirmed and probable cases were abstracted for housing status, demographics, and risk factors such as the use of illicit substances. We attempted to interview all patients, both PEH and non-PEH, with a standardised shigellosis questionnaire. An additional hypothesis-generating questionnaire was administered to 10 hospitalised patients to investigate specific risk factors among PEH, including housing and eating locations, sanitation practices, illicit substance use practices, and water sources. If a completed interview was not available, information from medical records was used.

### Environmental comparison

We compared National Oceanic and Atmospheric Administration (NOAA) data on inches of precipitation in the San Diego area during August–December 2021 to 30-year historical averages and distributions for those months.

### Laboratory and genomic methods

CIDT by gastrointestinal multiplex PCR panel was performed in hospital laboratories on stool specimens to characterise the presence of *Shigella* spp.; specimens were cultured for speciation and tested for antimicrobial resistance in samples that had viable organism for culture. WGS was requested for all culturable samples and was performed at SDC Public Health Laboratory using the PulseNet™ Standard Operating Protocol for the MiSeq platform (Illumina, San Diego, CA). Sequences were classified by core genome multilocus sequence typing (cgMLST) and compared with sequences available on PulseNet using the National Center for Biotechnology Information Pathogen Detection Isolates Browser [12]. The relatedness of sequences was assessed using the number of alleles with differing sequences [13]. WGS with 10 or fewer allele differences collected within a 60-day time frame was considered related or matched within a cluster of three or more sequences, where two or more sequences also had five or fewer allele difference; sequences not meeting this definition were included in a cluster only after consultation with CDC PulseNet [14].

### Statistical and graphical methods

We used descriptive statistics to compare patient demographics, reported exposures, and geographic location. Maps (ArcGIS version 10.8.1, Esri) of locations used for sanitation, water sources, sleeping, and other daily activities reported by patients were used to guide locations for additional handwashing stations and increased public restroom cleaning. WGS results were categorised by genetic cluster. Moran’s I method [15] was used to assess for spatial correlation of genetic clusters based on coordinates of reported patient primary living area, with a result of p < 0.05 considered statistically significant.

### Ethics

This activity was reviewed by CDC and was conducted consistent with applicable federal law and CDC policy.^1^

## Results

**Figure 1.**
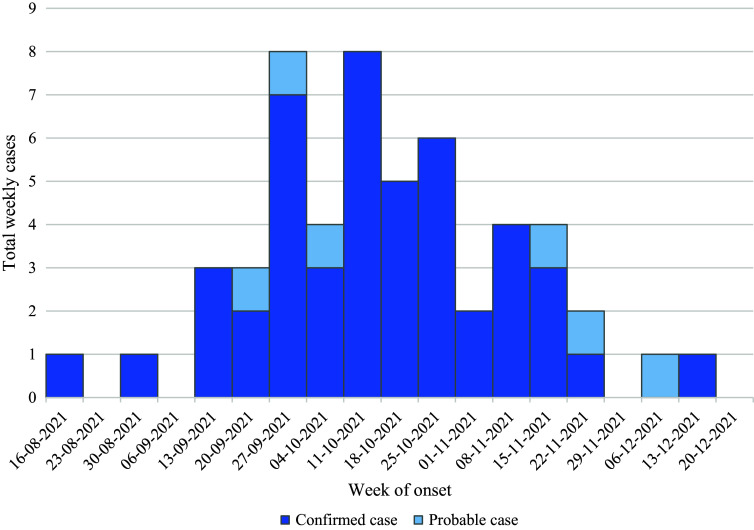
Shigellosis cases in persons experiencing homelessness by week of illness onset* (*N* = 53). *When onset date is unavailable, specimen collection date is used.

Fifty-three cases of shigellosis among PEH were identified with onset dates during August–December 2021 (Figure 1). Of these, 47 (89%) met the confirmed case definition with culture-confirmed *S. sonnei*, and 6 (11%) were probable cases positive for *Shigella* spp. by CIDT. Median age of patients was 46 years (range: 19–70 years) (Table 1). A total of 32 (60%) patients identified themselves as cisgender male, 19 (36%) as cisgender female, and 2 (4%) as transgender female (Table 1). Fifty-one (96%) patients visited an emergency department. Thirty-four (64%) patients were hospitalised for their shigellosis infection; none died. One other patient was already hospitalised for another illness and tested positive for Shigella after diarrhoea was noted during the hospital stay. Median hospital length of stay was three days (interquartile range: 2–5 days). Five patients were provided isolation housing in local hotels during their infectious periods to avoid discharging still-infectious patients to communal living settings, including shelters or encampments.

**Table 1.** Selected demographics and characteristics of persons experiencing homelessness who met the case definition (*N* = 53)

Characteristic	No. (%)
Gender	
Cis male	32 (60.4)
Cis female	19 (35.8)
Trans male	0
Trans female	2 (3.8)
Age (years)	
<18	0
18–44	24 (45.3)
45–64	25 (47.2)
≥65	4 (7.5)
Reported sexual partnerships	
Men who have sex with men (MSM)^a^	2 (3.8)
Other than MSM	21 (39.6)
Unknown or declined to answer	30 (56.6)
Reported illicit drug use	
Methamphetamine^b^	29 (54.7)
Fentanyl	1 (1.9)
Methamphetamine and fentanyl or other opioid^b^	8 (15.1)
Cannabis only	3 (5.7)
Unspecified	3 (5.7)
No illicit drug use reported	2 (3.8)
Unknown	7 (13.2)
Reported intravenous drug use	
Yes	7 (13.2)
No	46 (86.8)
Required hospitalisation because of shigellosis infection	
Yes	34 (64.2)
No	18 (34.0)
Unknown	1 (1.9)
Case classification	
Confirmed	47 (88.7)
Probable	6 (11.3)
Sample sequence result	
National cluster 2108MLJ16–1	25 (47.2)
Local cluster	20 (37.7)
No sequence performed	8 (15.1)

a Classified based on natal sex of patient and reported partners.

b With or without concomitant cannabis use.

Twenty (38%) of 53 patients completed interviews using the standardised shigellosis questionnaire, and 10 (19%) also completed detailed hypothesis-generating questionnaires. The remaining 33 patients (62%) completed partial interviews (6 (11%)) or could not be reached (27 (51%)). Of 46 patients for whom information on illicit substance use could be obtained, 44 (96%) used illicit substances. Of these 44, 37 (84%) used methamphetamine (Table 1). Of 16 patients reporting a route of methamphetamine use, 9 (56%) reported smoking or snorting methamphetamine without any intravenous use; the remaining 7 (44%) reported intravenous use. Of those using intravenous methamphetamine, 3 completed a detailed interview, and all 3 reported using bottled water to prepare methamphetamine injections. Of 22 patients providing a sexual history, 20 (91%) reported partners of a different sex than their own and 2 (9%) reported partners of the same sex or more than one sex.

**Figure 2.**
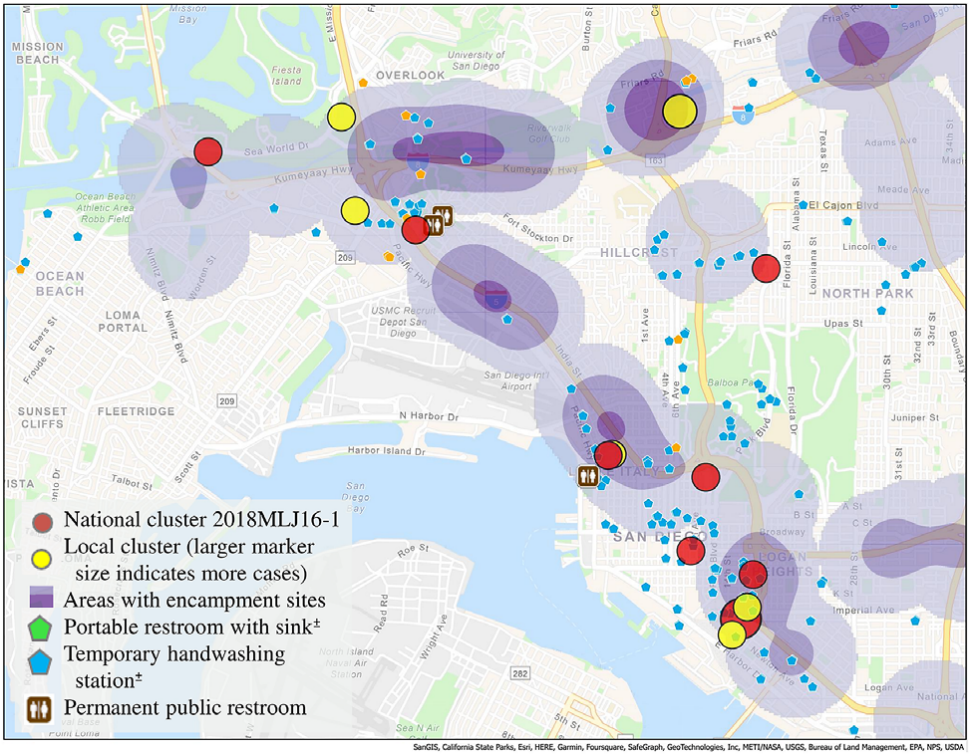
Geographic location* of patient living areas in San Diego City by whole genome sequencing cluster, with sanitation facilities and approximate densities of encampment sites. *Geographic locations of patient living locations are approximated to decrease identifiability. ^†^Areas with higher density of encampments are indicated in deeper purple. ^±^Added or relocated in response to this outbreak.

Epidemiologic investigation found geographic clustering of patient living areas near downtown San Diego City; geographic clusters did not correspond to WGS clusters (Figure 2). Reported living areas included shelters serving PEH, encampments, and unsheltered public parks, many of which were over a mile from permanent public restrooms. Multiple cases of *S. sonnei* were also identified among non-PEH during this time and had sequences matching one of the clusters associated with the outbreak, including two cases likely acquired at a restaurant in downtown San Diego that also provided restroom access and food to PEH.

**Figure 3.**
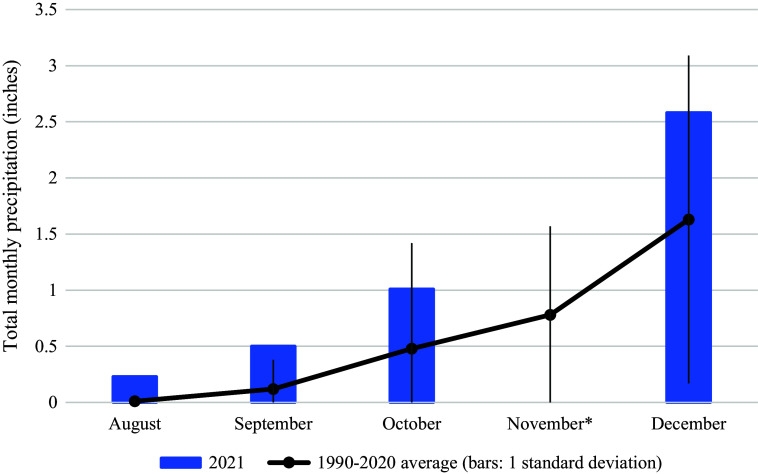
Total monthly precipitation in inches, National Oceanic and Atmospheric Administration, San Diego area, August–December 2021, compared with August–December 1990–2020. *The footnote with this figure is to indicate that there was no rainfall in November (refers to the asterisk on the x axis).

Of the 10 PEH interviewed with the hypothesis-generating questionnaire, 7 reported exclusively using bottled water or accessing clean water at fast food restaurants offering free cups of water, and 3 reported using public water fountains or taps. No common sources of food or water were identified. Five patients identified limitations in their access to restrooms, including a lack of paper towels or soap in public facilities or routinely using bushes or other natural features as restrooms. Precipitation during August–October 2021 was higher than 30-year averages for the NOAA San Diego area, with a Z-score indicating that August and September rainfall exceeded the 95^th^ percentile (Figure 3).

A total of 45 (85%) cultured samples had sufficient isolates for WGS. Of these sequenced isolates, 25 (56%) matched an ongoing U.S. *S. sonnei* cluster, 2108MLJ16–1, with 0–17 allele differences from each other. National cluster 2108MLJ16–1 had 122 cases in 12 states as of 13 December 2021, with isolation dates occurring as early as June 2021. The majority of isolates reported in this national cluster were from California residents (108; 89%), with 76 (62%) in SDC. Among 25 outbreak samples in this cluster, 12 (48%) were resistant to trimethoprim-sulfamethoxazole (TMP-SMX), 2 (8%) were resistant to TMP-SMX and ciprofloxacin, 2 (8%) were resistant to TMP-SMX and ampicillin, 3 (12%) had no detected antibiotic resistance, and resistance was unknown for 6 (24%).

Comparison of WGS identified an additional distinct cluster of *S. sonnei* isolates within 0–7 alleles of each other among 30 SDC residents with collection dates as early as July 2021. These isolates differed by approximately 29 alleles from the national cluster 2108MLJ16–1. Of 30 patients within this local cluster, 20 were part of the outbreak among PEH. Among 20 outbreak samples, 11 (55%) were resistant to TMP-SMX, 1 (5%) was resistant to TMP-SMX and ampicillin, 1 (5%) was resistant to azithromycin, and resistance was unknown for 7 (35%); no isolates were found to be extensively antibiotic-resistant as all were susceptible to at least some commonly recommended empiric and alternative antibiotics. No clear geographic clustering was observed among location data collected from PEH when separated by genetic strain of infection; a Moran’s I test did not suggest spatial correlation (*P* = 0.58).

### Interventions

**Figure 4.**
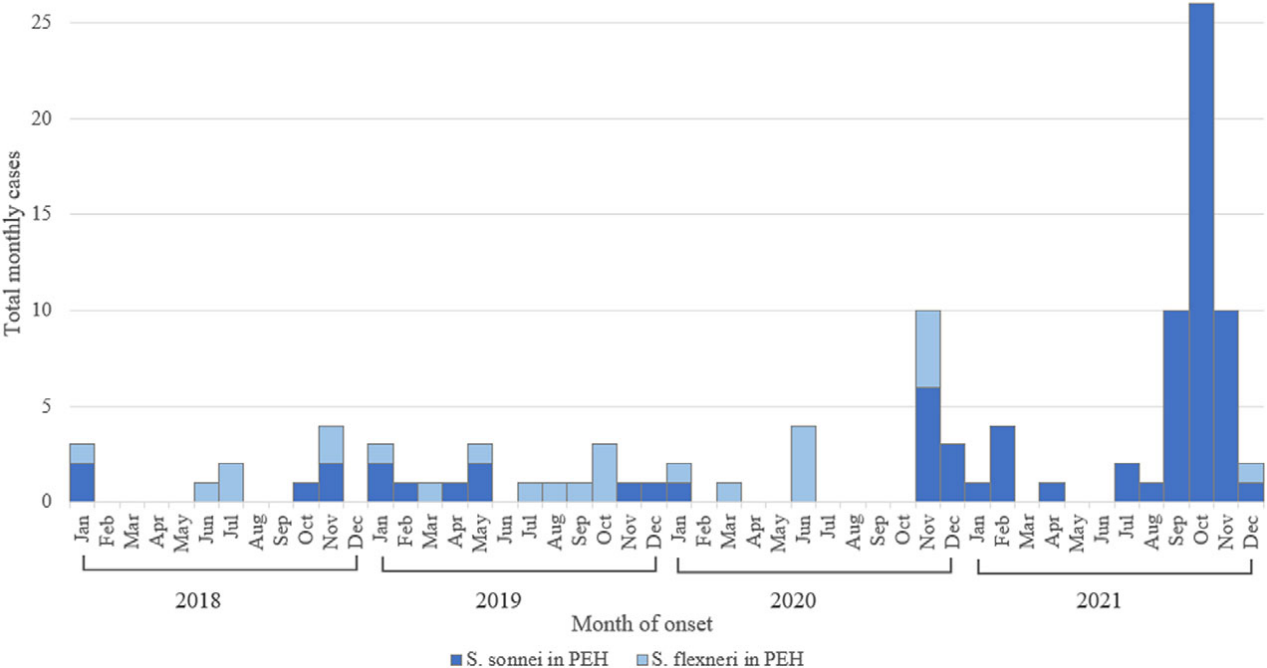
*Shigella sonnei* and *Shigella flexneri* cases in persons experiencing homelessness by month, San Diego County, 2018–2021*. *When onset date is not available, specimen collection date is used.

SDC, in partnership with San Diego City, deployed or relocated 99 portable handwashing stations to areas with the highest concentration of detected cases in PEH, beginning in mid-October. Public restrooms in parks and other public areas were cleaned and stocked more regularly with soap and toilet paper. Portable restrooms were also placed near encampments and other areas frequented by PEH. Sidewalk sanitisation in downtown San Diego City and other affected areas was increased from twice weekly to daily. Outreach teams distributed approximately 1,000 hygiene kits (including hand sanitiser, bottled water, and hand wipes) weekly along with additional information on preventing *Shigella* transmission and shelter resources. Messaging was provided to area shelters to educate clients on the importance of handwashing and disease prevention. Temporary private lodging in hotels was provided by SDC for PEH as needed during infectious periods to prevent transmission in congregate settings such as shelters or camps. *S. sonnei* cases decreased to expected frequencies after these interventions (Figure 4). The outbreak was declared over on 14 January 2022 [16].

## Discussion

A lack of public sanitation facilities and resultant person-to-person spread was the most likely contributing factor identified during this outbreak investigation, which involved two genetically distinct strains of *S. sonnei.* This finding is supported by patient reports of difficulty accessing sanitation facilities and known infectiousness of *Shigella*, especially in settings with inadequate sanitation and hygiene. Closure of some public restrooms and decreased maintenance of others in response to the COVID-19 pandemic might have contributed to *Shigella* transmission. We did not identify a single point source, such as a common food source, water source, illicit drug use pattern, or other clear shared exposure for the two genetic clusters. This suggests the outbreak is more likely attributed to adequate transmission opportunities for this highly contagious bacterium, rather than a single strain of bacteria having acquired uniquely transmissible characteristics. Ample opportunities for transmission were likely presented within encampments and other places in which access to clean water and restrooms was limited.

A relatively high proportion of patients associated with this outbreak were hospitalised (34/53, 64%). The admission rate during this outbreak might be higher than that in populations not experiencing homelessness for multiple reasons. Most cases were identified only when PEH with shigellosis visited an emergency department or were hospitalised, which likely indicates that milder infections were missed; the hospitalisation rate and case total reported here reflect only detected cases. Case-finding efforts included communications with San Diego physicians and emergency departments to be alert for shigellosis among PEH. Separately, public communications included handing out information about shigellosis to PEH in shelters and other locations, encouraging them to isolate and seek care for symptoms. However, the possibility exists that not all PEH with diarrhoeal illness sought care. Possible reasons for not seeking care might include a lack of a primary care physician or medical home, inability to get an appointment with primary care, lack of transportation, distrust of medical institutions, fear of incurring medical costs, or reluctance to visit an emergency department for a nonemergent illness. Additionally, hospital admission decisions consider social factors that include the ability of a patient to care for themselves, access to adequate sanitation facilities, and medical factors (e.g., severity of a patient’s illness). PEH might be more likely to be admitted to a hospital for reasons that include social factors. Although PEH are more likely than the general population to have chronic medical conditions and to experience health disparities or inadequate care for their medical conditions [17, 18], a minority of patients associated with this outbreak reported underlying medical conditions.

WGS demonstrated two distinct genetic clusters of *S. sonnei* within the outbreak; both were endemic strains detected repeatedly in SDC before this outbreak. In comparison with pathogens prone to mutation, including certain RNA viruses for which transmission events can sometimes be inferred from genomic sequencing results, *Shigella* has a relatively conserved genome and low transmission divergence [19]. The window for genomic mutation is long enough that reintroduction could not be excluded during this investigation, so it could not be concluded that the genomic clusters identified originated from a single source. Genetic clusters were not found to be associated with distinct geographic clusters, nor were other unique exposures such as common food or water sources or injection drug use practices identified during epidemiologic investigation. Although individual sequences were not distinct enough to assist with tracing disease transmission between individuals, WGS did help confirm that sequences associated with this outbreak were genomically similar to the two strains previously identified circulating in the community.

The COVID-19 pandemic unearthed and exacerbated existing inequities, including those affecting California’s large and rapidly growing population of PEH. In SDC, the estimated total number of PEH increased by 10% during January 2020–January 2022 [20]. At the same time, risks associated with SARS-CoV-2 transmission led to changes in service provision across the United States, from restricting access to public restrooms and other sanitation facilities to reducing capacity limits in some shelters. Although these measures might have had protective effects against SARS-CoV-2 transmission among PEH, they might inadvertently have led to other health risks. This includes diversion from shelters to encampments with limited sanitation facilities, increased food insecurity, increased exposure to interpersonal violence, and increased exposure to inclement weather [21]. Climate change could also exacerbate public health challenges in ways that have the most substantial effects on PEH, such as changing precipitation patterns thought to influence the risk of *Shigella* transmission among PEH [7] or wildfires or other disasters aggravated by climate change decreasing available housing in a community [22]. Higher than usual rainfall in the San Diego area during August–October 2021 might have contributed to poorer sanitation and more crowding in shelters and encampments during those months, whereas an unusually dry November 2021 might have assisted mitigation efforts and helped slow transmission. Although rainfall was subsequently above average during December 2021, efforts to improve sanitation and hygiene were well underway before that time. This suggests that the effects of increased rainfall on disease transmission could be mitigated with public sanitation and hygiene improvements.

Health concerns among PEH are also not solely confined to that population, because communicable disease in one group in a community often affects the health of others. For example, during a 2017 outbreak of hepatitis A, homelessness was an independent risk factor for acquiring hepatitis A, but 37% of those who acquired hepatitis A were not homeless [23]. In the outbreak reported here, there are cases of two non-PEH patients with similar sequencing results that might have been related to fomite transmission in a restaurant, which suggests transmission between PEH and non-PEH took place during this outbreak. Illnesses affecting PEH might have effects on non-PEH, underscoring the interdependence of community health, especially the importance of ensuring adequate sanitation facilities are readily accessible by all members of a community regardless of housing status.

The finding of multiple circulating endemic strains within this outbreak highlights that inadequate sanitation presents a myriad of opportunities for transmission. Inadequate access to clean restrooms and running water among interviewed PEH were common risk factors identified and are likely to have contributed to transmission. Expanding public access to sanitation facilities, coupled with intensive interventions among PEH to stop transmission, such as distributing hygiene kits and providing isolation housing, likely helped end this outbreak. Continuing to ensure adequate sanitation facilities are provided and accessible for all members of the community is crucial for preventing future community outbreaks of enteric disease.

The findings of this report are subject to at least three limitations. First, some patients declined to or were unable to complete a full interview. Therefore, epidemiologic information for this outbreak could be incomplete. Second, cases classified as probable had CIDT results but no specimen available for WGS, so these cases could not be considered when assessing for geographic clustering by strain. Finally, the two non-PEH with *Shigella* that were identified as having been in or near the same location as PEH might have other risk factors besides fomite transmission to explain their infection that were not identified during this investigation. However, because another source was not identified, transmission prevention efforts focused on hygiene interventions in the PEH population.

## Recommendations

Both people experiencing homelessness and those not experiencing homelessness require access to sanitation facilities to prevent transmission of infections such as shigellosis [24]. Changes to public sanitation facilities, including pandemic response measures, should ensure that adequate, readily accessible alternatives for the populations who rely on these facilities are provided. Individuals should be educated on key behaviours to prevent transmission of *Shigella* and other faecal–oral diseases, including handwashing with soap and water; avoiding swimming, sex, and food preparation for others while symptomatic with diarrhoea; and avoiding swallowing water from ponds, lakes, or swimming pools. Prevention and healthcare promotion messages should be tailored for specific populations based on those most affected by an outbreak, such as PEH. Ensuring continuous public access to clean water and adequate sanitation facilities, particularly in areas where many people depend on public facilities to meet their needs, is important to prevent shigellosis among PEH and to protect the health of the entire community.

## Data Availability

Data supporting the findings of this analysis are available within the article; additional de-identified data are available from the corresponding author upon reasonable request.

## Footnotes

1 45 C.F.R. part 46, 21 C.F.R. part 56; 42 U.S.C. Sect. 241(d); 5 U.S.C. Sect. 552a; 44 U.S.C. Sect. 3,501 et seq.
